# Mesencephalic astrocyte-derived neurotrophic factor reprograms macrophages to ameliorate acetaminophen-induced acute liver injury via p38 MAPK pathway

**DOI:** 10.1038/s41419-022-04555-9

**Published:** 2022-02-02

**Authors:** Xin Hou, Qi Liu, Yimin Gao, Liang Yong, Huiyuan Xie, Wenting Li, Yuping Zhou, Jun Liu, Lijie Feng, Long Xu, Yuxian Shen, Hua Wang

**Affiliations:** 1 The Affiliated Hospital of Medical School, Ningbo University, Ningbo, China; 2 School of Medicine, Ningbo University, Ningbo, China; 3 School of Basic Medical Sciences, Anhui Medical University, Hefei, China; 4 The First Affiliated Hospital, Division of Life Sciences and Medicine, University of Science and Technology of China, Hefei, China; 5 Department of Oncology, the First Affiliated Hospital of Anhui Medical University, Hefei, China

**Keywords:** Monocytes and macrophages, Hepatitis

## Abstract

Acetaminophen (APAP)-induced liver injury (AILI) is the most frequent cause of acute liver failure; but the underlying mechanisms still remain obscure. Macrophages and endoplasmic reticulum (ER) stress play an important role in the pathogenesis of AILI. Mesencephalic astrocyte-derived neurotrophic factor (MANF) is a newly identified 18-kDa soluble protein, whose expression and secretion are stimulated by ER stress. To investigate the role of myeloid cell MANF in the pathogenesis of AILI, we assayed serum and liver samples from AILI model mice and patients with drug-induced liver injury (DILI). We demonstrated that the levels of MANF were elevated in patients with DILI and in mice with AILI. Moreover, myeloid-specific MANF knockout mice were generated and used. It was observed that a delayed liver recovery from myeloid-specific MANF gene knockout mice following APAP overdose compared to that from wild-type mice. MANF deficiency in myeloid cells resulted in increased infiltrating monocyte-derived macrophages (MoMFs) but reduced restorative Ly6C^low^ macrophages after APAP treatment. MANF supplementation increased restorative Ly6C^low^ macrophages and subsequently alleviated liver injury. Moreover, MANF could enhance IL-10 expression and phagocytosis in macrophages via p38 MAPK pathway. Altogether, MANF seems to be a critical immune modulator in promoting liver repair via reducing and reprogramming MoMFs. MANF perhaps promoted the phenotype conversion of pro-inflammatory MoMFs to pro-restorative Ly6C^low^ MoMFs via p38 MAPK pathway, particularly through enhancing IL-10 and phagocytosis.

## Introduction

Drug-induced liver injury (DILI) is one of the major causes of liver diseases around the world. In Western society, the main cause of acute liver injury is acetaminophen (APAP) overdose [1]. APAP is metabolized by hepatic cytochrome P450 enzymes (mainly CYP2E1) into a reactive metabolite N-acetyl-p-benzoquinoneimine (NAPQI) which is detoxified by glutathione (GSH) [2]. Excessive formation of NAPQI causes depletion of hepatic glutathione in APAP overdose and subsequently leads to oxidative stress and hepatocyte damage [3]. Currently, N-acetylcysteine is the only clinically approved antidote for APAP overdose patients. However, the efficacy of N-acetylcysteine dramatically decreases 8 h after APAP ingestion [4, 5]. Therefore, the development of new approaches to treat APAP-induced liver failure, particularly at the late phase of injury is required.

Initial NAPQI-induced direct hepatocyte damage leads to the release of damage-associated molecular patterns (DAMP), which activates cytokines and chemokines formation in resident Kupffer cells for recruitment of neutrophils and circulating monocytes [6, 7]. CCL2 (MCP-1) provokes recruitment of circulating CCR2^+^ Ly-6C^hi^ monocytes into injured liver [8]. These monocyte-derived macrophages (MoMFs) represent the main liver macrophage population after APAP injury, and play contradictory roles in APAP-induced liver injury (AILI) [9]. It has been demonstrated that Ly-6C^hi^ macrophages have a pro-inflammatory phenotype reflected by the expression of inflammatory cytokines. Hence, antagonists against CCL2 or CCR2 ameliorates the early but not the late phase of AILI via reducing the recruitment of monocytes [10, 11]. Interestingly, other studies report that these Ly-6C^hi^ monocytes differentiate into Ly-6C^low^ restorative macrophages at the site of injury. Ly-6C^low^ macrophages express anti-inflammatory cytokines, regenerative growth factors and matrix degrading metalloproteinase to promote resolution from injury [12–15]. Signals derived from the hepatic microenvironment (e.g. macrophage colony-stimulating factor 1 (CSF1), IL-6, phagocytosis of cellular debris) may promote the phenotypic and functional transition of macrophages [16–18].

Mesencephalic astrocyte-derived neurotrophic factor (MANF), a new evolutionary conserved neurotrophic factor, is widely expressed in neuronal and non-neuronal tissues [19, 20]. MANF expression and secretion are regulated by endoplasmic reticulum (ER) stress and protects against various ER stress-induced damage [21, 22]. Recent studies demonstrate that MANF has an autocrine immunomodulatory function in macrophages, which is essential for its neuroprotection and tissue repair in retina and liver [23]. However, the underlying mechanism of MANF in signaling remains elusive. In this study, we identified MANF to be a protective mediator that promoted resolution of AILI by regulating monocyte infiltration and triggering macrophages conversion into a reparative phenotype. Mechanistically, we demonstrated that MANF promoted the production of IL-10 and phagocytosis in macrophages though p38 kinase. Treatment of mice with recombinant human MANF (rhMANF) was sufficient to facilitate liver repair, which suggested that MANF may serve as a promising therapeutic strategy for APAP overdose and other liver injuries.

## Materials and methods

### Human samples and ethics statement

Liver tissues and blood samples of patients with drug-induced liver injury were collected from the First Affiliated Hospital of University and Science and Technology in this study (*n* = 24). In addition, 24 healthy blood samples were included as a control group. Normal liver samples were obtained from surgical resections of uninvolved liver tissues of angioma patients (*n* = 20). Informed consent was obtained from all subjects. This study was approved by the Ethics Committee of Anhui Medical University and the First Affiliated Hospital of University and Science and Technology.

### Animals

*Manf*^flox/flox^ mice with C57BL/6 J background were kindly provided by Professor Jia Luo of Kentucky University, USA. *Manf*^Hep-/-^ mice and *Manf*^Mye-/-^ mice were generated by a few steps including crossing *Manf*^flox/flox^ mice with Albumin Cre mice and Lysosome Cre mice, respectively. All mice were maintained in a specific pathogen-free facility. All animal experiments were approved by the Institutional Animal Care and Use Committee at Anhui University.

### Animal treatment

To induce acute liver injury, fresh solutions of APAP (Sangong, Shanghai, China) were prepared immediately before use by dissolving the compound in warmed PBS. After overnight fasting, 8–10 week old male mice were injected with APAP 300 (mg/kg) intraperitoneally. For the treatment, some of the mice were intravenously injected with recombinant human MANF (1.5 mg/kg, SinoBiological, Beijing, China) or recombinant mouse IL-10 (1 μg/mouse, Biolegend, San Diego, CA, USA) 0.5 h after APAP administration.

### Serum ALT assay

Serum ALT levels were measured using an ALT assay kit (Nanjing Jiancheng Bioengineering Institute) according to the manufacturer’s instructions.

### Serum MANF assay

Serum MANF levels were measured by using the MANF ELISA Kit 96 T (Reddot Biotech Inc) according to the manufacturer’s instructions.

### Total RNA isolation and real-time quantitative PCR

Total RNA was purified from samples using TRIzol reagents (Invitrogen, Carlsbad, CA, USA) according to the manufacturer’s instructions. First strand cDNA was synthesized using the PrimeScript^TM^ RT reagent Kit (Perfect Real Time) (TaKaRa). Quantitative PCR was performed using SYBR Premix Ex TaqTM II (Tli RNaseH Plus) (TaKaRa) on a StepOnePlus Real-Time PCR System (Applied Biosystems, Foster City, CA, USA). Each test was repeated three times and the 2^−ΔΔ^ Ct method was used to calculate the expression of mRNA with β-actin as an internal control. The primer sequences are listed in Supplementary Table 1.

### Cell isolation

Hepatic leukocytes were isolated as described previously [24]. Briefly, liver tissues were minced and sieved through a 70 μm filter. Hepatic leukocytes were purified by centrifugation on a 40% Percoll gradient (GE Healthcare), and red blood cells were lysed by RBC lysis buffer. Collected leukocytes were further enriched using PE-anti-F4/80 (BD Pharmingen, Cat# 565410) and anti-PE microbeads (Miltenyi Biotec) to isolate macrophages. Hepatocytes were isolated as described previously [25]. Briefly, the liver was perfused with a solution of EGTA and digested with a 0.075% collagenase solution. The viable hepatocytes were separated by 40% Percoll solution with centrifugation at 420 × *g* for 10 min at 4 °C.

### Histology assay

Liver tissues were fixed in 10% formalin and paraffin-embedded following standard procedure. Embedded liver tissues were sectioned, and stained with hematoxylin and eosin (H&E) or subjected to immunohistochemical staining using anti-MANF antibody (Abcam, Cambridge, MA, USA, ab67271), anti-Cyp2E1 antibody (Abcam, Cambridge, MA, USA, ab28146) according to the manufacturers’ protocols. The necrosis areas were outlined according to the cellular structure and color. The percentages of necrosis areas were averaged from 5 to 7 random view fields of each section (×200 magnification) using ImageJ software.

Immunofluorescence staining was performed on frozen sections with rabbit anti-mouse F4/80 (1/200 dilution, Cell Signaling Technology, Danvers, MA,70076), FITC conjugated goat anti-rabbit IgG (H + L) (1/100 dilution, ZSGB-BIO, ZF0311), mouse anti-mouse MANF antibody, Rhodamine conjugated goat anti-mouse IgG (H + L) (1/100 dilution, ZSGB-BIO, ZF0313), and 4′, 6′-diamino-2-phenylindole (DAPI). The images were obtained by using Leica TCS SP8 confocal microscope.

### Flow cytometry analysis

The following fluorochrome-conjugated monoclonal antibodies were used in this study: anti-mouse PE/Cy7-conjugated CD45 (Cat# 940335), APC-conjugated Ly6G (Cat# 560599), PE-conjugated CD11b (Cat# 553311), PE-conjugated CD19 (Cat# 553786), and APC/Cy7-conjugated NK1.1(Cat# 560618) (BD Biosciences, San Diego, CA, USA). PerCP/Cy5.5-conjugated F4/80 (Cat# 157317), APC/Cy7-conjugated Ly6C (Cat# 128025), FITC-conjugated CD14 (Cat# 123308), (Biolegend, San Diego, CA, USA), and PE/Cy7-conjugated CD3 (Cat# 25-0031-82) (eBioscience, San Diego, CA, USA). Cells were blocked with FcR blocker (BD Pharmingen) and then incubated with the indicated antibodies under standard protocols. All samples were analyzed using flow cytometry (FACSVerse system, BD Biosciences) with FlowJo (version 7.6.1) software.

### Western blot

Harvested cells were homogenized in RIPA buffer (50 mM Tris-HCl, pH 7.4, 0.1% SDS, 1% NP40, 0.5% sodium deoxycholate, 150 mM NaCl, 1 mM EDTA) supplemented with protease inhibitor cocktail (Roche, Indianapolis, IN, USA). Proteins were separated on 10% SDS-PAGE gels and were then transferred to NC membranes (Beyotime, Shanghai, China). Membranes were blocked 2 h in 5% nonfat milk and probed with the following primary antibodies against: P-AKT (Cat# 4060)/AKT (Cat# 4685), P-JNK (Cat# 9255)/JNK (Cat# 9252), P-ERK (Cat# 4370)/ERK (Cat# 4695), p-p38(Cat# 4511)/p38(Cat# 8690) (Cell Signaling Technology Inc, Beverly, MA, USA). All membranes were subsequently incubated for 2 h with Peroxidase-conjugated Goat anti-rabbit IgG (ZB-2301) (ZSGB-BIO, Beijing, China). Protein bands were visualized with SuperSignal West Femto Maximum Sensitivity Substrate (Thermo Scientific, Rockford, IL, USA). Antibody concentrations used were based on the manufacture’s recommendations.

### Cell treatment with rhMANF or inhibitors

THP-1 cells and primary peritoneal macrophages were cultured in RPMI1640 media supplemented with 10% fetal bovine serum and penicillin-streptomycin. Cells were pretreated with various MAPK inhibitors or AKT inhibitor for 1 h, followed by rhMANF (2 μg/mL) treatment for 5 h. The concentrations of the inhibitors used were as follows: c-Jun amino terminal kinase (JNK) inhibitor (20 μM SP600125, sigma S5567), p38 inhibitor (20 μM SB203580, sigma S8307), and ERK inhibitor (20 μM U0126, sigma U120), AKT inhibitor (20 μM MK-2206 dihydrochloride, TargetMol T1952).

### Phagocytosis assays

For detecting phagocytosis of macrophages, mouse primary hepatocytes or human hepatocytes (LO2 cells) were treated with 20 μM dexamethasone for 24 h and then labeled with 1 μM CFSE for 15 min at 37 °C. Mouse peritoneal macrophages, liver MoMFs (isolated from the same mouse as primary hepatocytes), or human monocytes (THP-1 cells) were challenged with CFSE-labeled apoptotic hepatocytes for 1 h at a 1:5 ratio at 37 °C or 4 °C. The cells were washed and stained with cell surface markers. Stained cells were analyzed by flow cytometry.

### Statistical analysis

Data are expressed as the means ± SD and were analyzed using GraphPad Prism software (v. 8.0a; GraphPad Software, La Jolla, CA, USA). The variance was similar between the groups that were being statistically compared. Comparisons between two groups were performed using an unpaired Student’s *t-*test. Comparisons among multiple groups were performed using two-way ANOVA followed by Tukey’s post hoc test. *P* < 0.05 were considered significant. Sample sizes of all experiments were predetermined from preliminary experiments or from reports in the literature. No sample was excluded from the analyses. All mice used in this study were chosen randomly. Investigators were not blinded to the group allocation during the experiment and outcome assessment.

## Results

### Increased MANF expression in DILI patients and an AILI mouse model

**Fig. 1:**
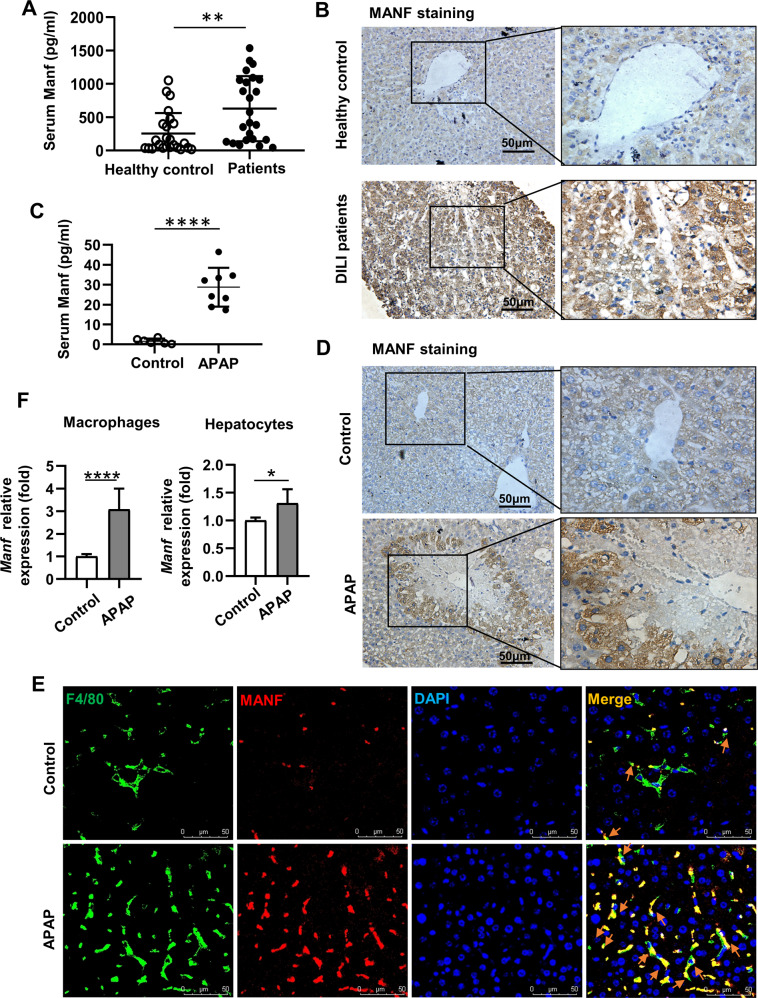
Increased MANF expression in drug-induced liver injury (DILI) patients and an AILI mouse model. **A** MANF was measured in serum samples of DILI patients (*n* = 24) and healthy controls (*n* = 24). **B** Representative immunohistochemical (IHC) images of hepatic MANF expression in DILI patients and healthy controls. **C** Serum levels of MANF in WT mice 24 h post APAP treatment were determined by ELISA. **D** Representative IHC images of hepatic MANF expression in mice 24 h after APAP treatment and in control mice. **E** Immunofluorescence staining for F4/80 (green), MANF (red) and DAPI for nuclei (blue) in WT mice liver tissue. Orange arrows point to F4/80^+^ macrophages with high expression of MANF. **F** Relative mRNA expression of MANF in macrophages and hepatocytes at 0 h (control) and 12 h after APAP treatment was determined by qPCR. Data are presented as mean ± SD and representative of at least 3 independent experiments. *n* = 6–8 per group. *P < 0 .05, **P < 0.01.

To investigate the role of MANF in DILI, we first examined MANF expression in DILI patients. As shown in Fig. 1A, B, serum levels and hepatic levels of MANF protein were markedly increased in DILI patients compared to healthy controls. A similar increase in MANF levels in the serum and liver tissues was observed in the AILI mouse model (Fig. 1C, D). Moreover, we performed an Immunofluorescence analysis to co-stain hepatic macrophages and hepatocytes with MANF. As illustrated in Fig. 1E, the expression of MANF in F4/80^+^ macrophages was increased significantly after APAP challenge, whereas the expression of MANF in hepatocytes was increased mildly (Supplementary Fig. S1). Consistent with the protein analysis results, the increase of *Manf* mRNA levels in macrophages (about 3-fold) was higher than that in hepatocytes (about 1.3-fold) (Fig. 1F).

### MANF in myeloid cells is critical for resolution of AILI

**Fig. 2:**
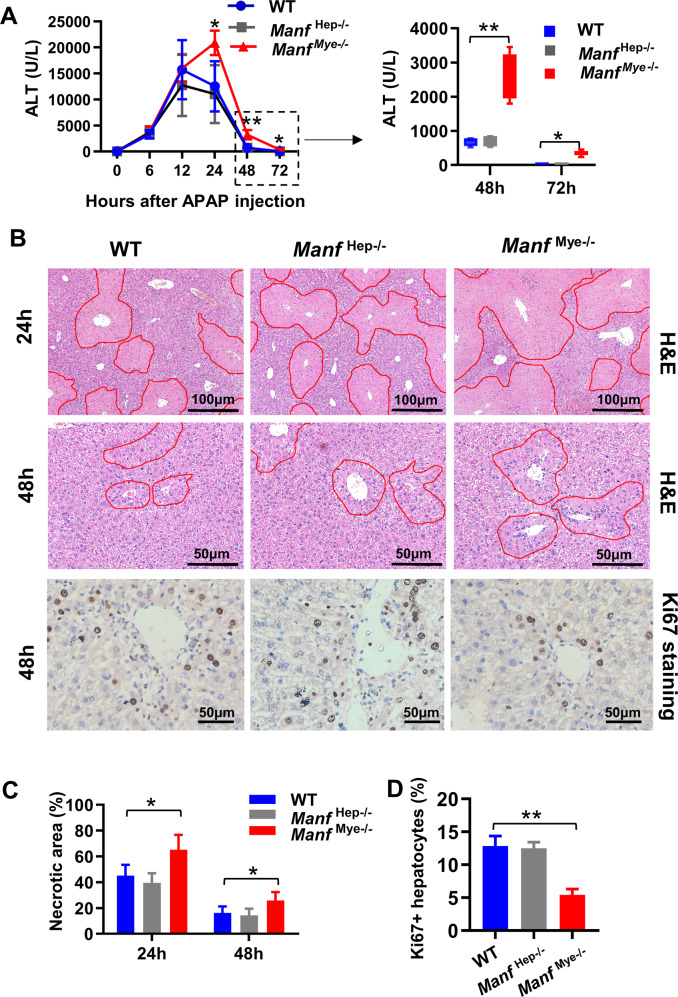
*Manf*^Mye-/-^mice display delayed liver recovery after APAP treatment. WT mice, *Manf*^Mye-/-^mice, and *Manf*^Hep-/-^ mice were intraperitoneally injected with APAP. **A** Serum ALT levels were measured after APAP treatment. **B** Representative H&E-staining and IHC staining for Ki67 in liver sections after APAP treatment. Necrotic areas were outlined using red lines. The percentages of necrosis area **C** and Ki67 **D** positive hepatocytes were quantified from 5 to 7 random view fields of each section (×200 magnification) using ImageJ software. *n* = 5–7/group, 3 independent experiments. Data are presented as mean ± SD. *P < 0.05, **P < 0.01.

To further dissect the role of MANF in hepatocytes and myeloid cells, we generated hepatocyte-specific *Manf* knockout mice (*Manf*^Hep-/-^), and myeloid-specific *Manf* knockout (*Manf*^Mye-/-^) mice, and treated these mice with APAP. Compared to WT mice, *Manf*^Mye-/-^ mice showed higher levels of serum alanine aminotransferase (ALT), larger areas of liver necrosis, and lower numbers of Ki67^+^ hepatocytes at the late phase post-APAP injection, whereas *Manf*^Hep-/-^ mice had comparable liver injury and regeneration to WT mice (Fig. 2A–D). In addition, deletion of MANF in hepatocytes did not significantly affect APAP-induced hepatocyte death in vitro (Supplementary Fig. S2). These results suggested that myeloid MANF played an important role in liver injury resolution after APAP treatment.

To study whether the delayed resolution of AILI in *Manf*^Mye-/-^ mice was attributed to alteration of APAP metabolism, we examined CYP2E1 and GSH expression in the liver. Our results showed that liver CYP2E1 protein expression and hepatic GSH levels were comparable between WT and *Manf*^Mye-/-^ mice after overnight fasting or nonfasting (Supplementary Fig. S3A–C).

### *Manf*^Mye-/-^mice have increased MoMFs but reduced restorative Ly6C^low^ MoMFs after APAP treatment

**Fig. 3:**
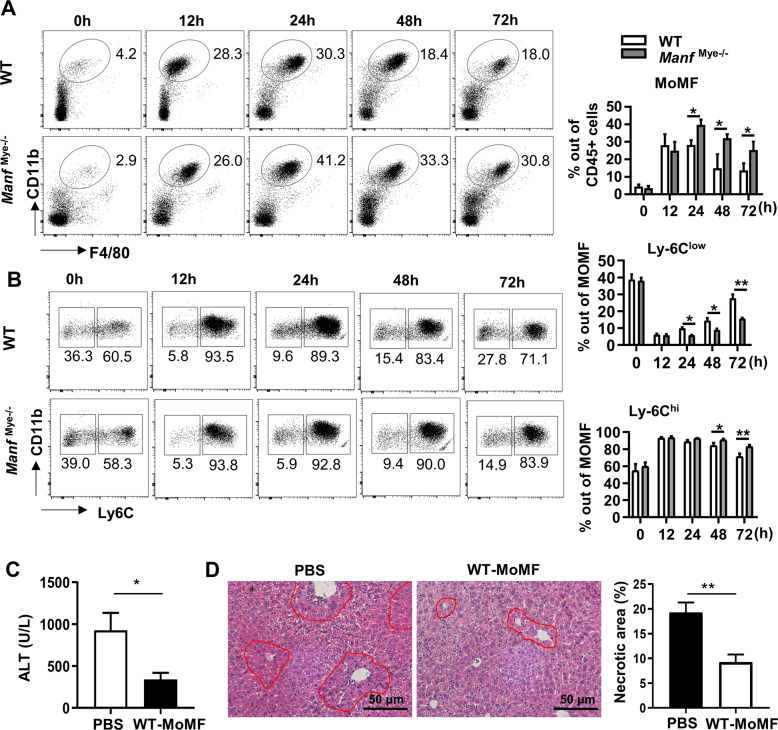
Increased MoMFs but decreased restorative Ly6C^low^ MoMFs in the liver of *Manf*^Mye-/-^mice after APAP treatment. WT and *Manf*^Mye-/-^mice were injected with APAP. **A** Representative flow cytometric plots and the statistical quantification of hepatic MoMFs (Ly6G^-^CD11b^high^ F4/80^intermediate^). **B** Representative flow cytometric plots and the statistical quantification of hepatic Ly-6C^hi^ and Ly-6C^low^ expressing MoMFs in WT and *Manf*^Mye-/-^mice. **C**, **D** MoMFs were isolated from WT mice at 72 h following APAP administration. *Manf*^Mye-/-^mice received (5 × 10^5^ cells) WT-derived MoMFs at the same time as APAP administration, and were sacrificed 48 h later. **C** Serum ALT levels were measured. **D** The percentage of necrotic areas in the liver was quantified. Data are presented as mean ± SD. *n* = 5–7, 3 independent experiments. *P < 0.05, **P < 0.01.

We then evaluated the populations of macrophages and neutrophils in the liver of *Manf*^Mye-/-^mice after APAP treatment by performing flow cytometric assays. The gating strategies for liver neutrophils and MoMFs were shown in Supplementary Fig. S4A. The percentages of neutrophils (CD45^+^CD11b^+^Ly6G^+^) (Supplementary Fig. S4B, C) and MoMFs (CD45^+^CD11b^high^Ly6G^-^F4/80^intermediate^) (Fig. 3A) were increased in the liver of *Manf*^Mye-/-^mice compared with WT mice at the late phase of APAP treatment. Next, we found that hepatic *Ccl2* and *Ccl3* levels were higher in *Manf*^Mye-/-^ mice after APAP treatment than those in WT mice (Supplementary Fig. S5A). Furthermore, *Manf*^Mye-/-^ mice showed increased monocytes in peripheral blood after APAP treatment compared to WT controls (Supplementary Fig. S5B, C). We also observed that monocytes from *Manf*^Mye-/-^ mice had a higher expression of CCR2 (Supplementary Fig. S5D, E). In addition, MANF deficiency did not influence the proliferation and survival of macrophages (Supplementary Fig. S6A–D). These results suggested that MANF limited monocyte influx by inhibiting the expression of *Ccl2* and *Ccl3* in the liver and the expression of CCR2 on monocytes.

Moreover, *Manf*^Mye-/-^mice had a lower percentage of Ly6C^low^ restorative MoMFs and a higher percentage of Ly6C^hi^ MoMFs between 24 and 72 h post-APAP application (Fig. 3B). To further confirm the protective role of Ly6C^low^ restorative MoMFs in AILI, we adoptively transferred Ly6C^low^ MoMFs from APAP-treated WT mice into *Manf*^Mye-/-^mice at the same time with APAP administration. The *Manf*^Mye-/-^mice that received Ly6C^low^ MoMFs showed decreases in ALT levels and hepatic necrosis (Fig. 3C, D).

### rhMANF enhances restorative macrophage percentage in vivo and facilitates liver recovery after acute liver injury

**Fig. 4:**
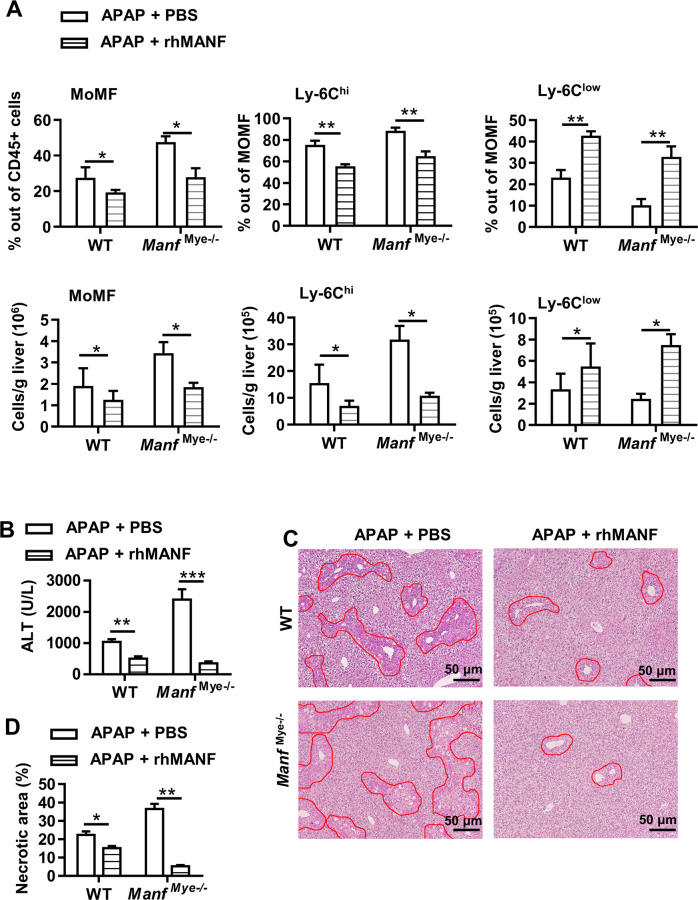
The rhMANF enhances the restorative macrophage phenotype in vivo and accelerates liver injury resolution. WT mice and *Manf*^Mye-/-^mice were intravenously injected with rhMANF (1.5 mg/kg) at the same time as APAP administration. Mice were sacrificed at 48 h after APAP administration. **A** The percentages and numbers of hepatic MoMFs, Ly-6C^hi^ expressing MoMFs, and Ly-6C^low^ expressing MoMFs were quantified by flow cytometric analysis. **B** Serum ALT activities were determined. **C** Representative H&E-stained liver sections. *n* = 5–7 sections/mouse. **D** Necrotic areas in the liver were quantified by ImageJ software. Data are presented as mean ± SD, *n* = 5–7 mice per group, 3 independent experiments.

The beneficial function of MANF in liver injury resolution prompted us to test whether MANF delivery is sufficient to elicit protective effects; thus, we treated WT mice and *Manf*^Mye-/-^mice with rhMANF at the same time as APAP administration. As shown in Fig. 4A, rhMANF treatment caused remarkable reductions in MoMFs and pro-inflammatory Ly-6C^hi^ MoMFs and a significant increase in restorative Ly-6C^low^ MoMFs during liver injury resolution (48 h after APAP administration). Critically, rhMANF treatment greatly decreased ALT levels (Supplementary Fig. S7A and Fig. 4B) and hepatic necrosis (Supplementary Fig. S7B, C and Fig. 4C, D) in both WT mice and *Manf*^Mye-/-^mice 24 h and 48 h after APAP administration. These data indicated that rhMANF induced macrophages to switch to a phenotype promoting liver injury resolution in vivo.

### The effect of myeloid cell-derived MANF on AILI depends on IL-10

**Fig. 5:**
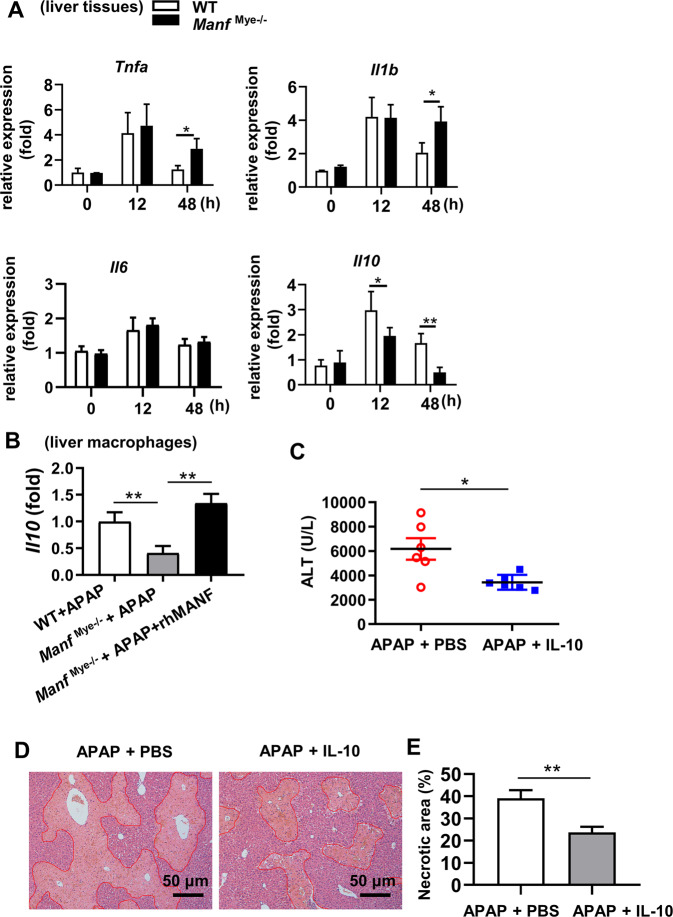
IL-10 decline was involved in delayed liver recovery in *Manf*^Mye-/-^mice after APAP treatment. **A** WT and *Manf*^Mye-/-^mice were injected with APAP. Hepatic mRNA expression levels of *Tnfa*, *Il1b*, *Il6*, and *Ifna* were measured by RT-qPCR. **B** WT mice and *Manf*^Mye-/-^mice were intravenously injected with PBS or rhMANF (1.5 mg/kg) at the same time as APAP administration. Hepatic macrophages were isolated from these mice at 24 h after APAP administration. *Il10* mRNA levels in hepatic macrophages were measured by RT-qPCR. **C**, **D** *Manf*^Mye-/-^mice were intravenously injected with recombinant IL-10 (1 μg/mouse) or PBS 0.5 h after APAP administration, and sacrificed at 24 h later. **C** Serum ALT levels were determined. **D** Representative H&E-stained liver sections. **E** Necrotic areas were quantified by ImageJ software. Data are presented as mean ± SD. *n* = 4–6 mice per group, 3 independent experiments. *P < 0.05, **P < 0.01.

Given that Ly6C^hi^ MoMFs secret inflammatory cytokines [10], whereas Ly6C^low^ MoMFs secrete anti-inflammatory cytokines [7], we examined cytokine expression in the liver of APAP-treated mice. RT-qPCR analysis showed significantly increased hepatic *Tnfa* and *Il1b* levels and decreased *Il10* levels in *Manf*^Mye-/-^ mice compared to WT mice after APAP treatment (Fig. 5A). Moreover, *Il10* levels in hepatic macrophages of APAP-treated *Manf*^Mye-/-^ mice were much lower than those in APAP-treated WT mice, and rhMANF dramatically up-regulated *Il10* levels in hepatic macrophages of *Manf*^Mye-/-^ mice (Fig. 5B). In addition, rhMANF increased *Il10* levels in the liver of both WT mice and *Manf*^Mye-/-^ mice, whereas had no effect on hepatic *Tnfa* and *Il1b* levels (Supplementary Fig. S8A).

It has been reported that macrophages secrete the anti-inflammatory molecule IL-10 that actively promote tissue repair [18, 26]. To explore whether delayed liver injury recovery is due to decreased IL-10 levels, we treated *Manf*^Mye-/-^ mice with IL-10 after APAP injection. As expected, treatment of mice with IL-10 significantly reduced liver injury at 24 h after APAP injection, which was demonstrated by reduced ALT levels (Fig. 5C) and less hepatocyte damage (Fig. 5D, E).

### MANF induces IL-10 expression in macrophages via p38 MAPK

**Fig. 6:**
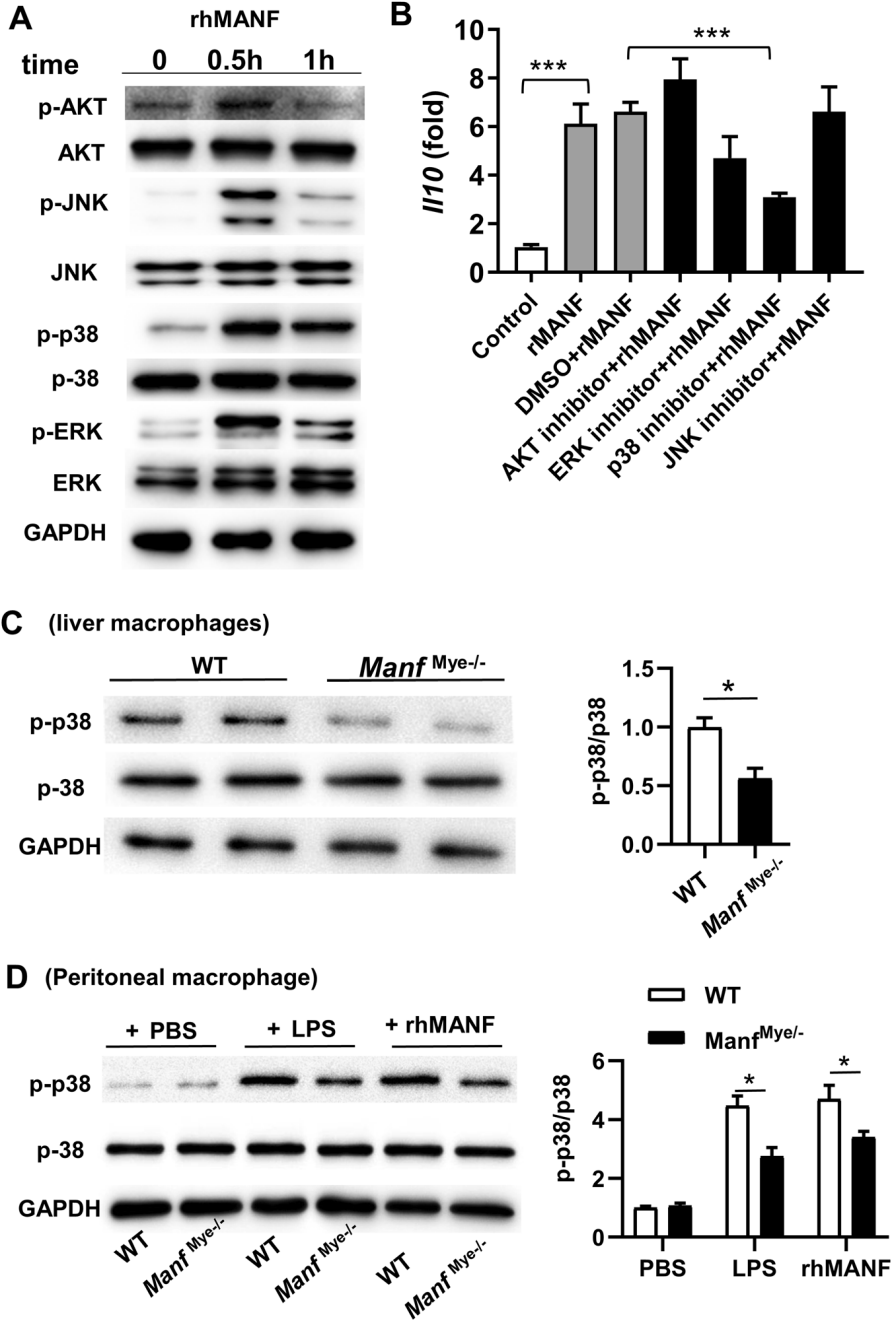
MANF induces IL-10 expression in macrophages via p38 signaling pathway. **A** Primary peritoneal macrophages of naive WT mice were treated with 2 μg/mL of rhMANF. Activation of various signaling pathways was measured by western blotting. **B** Primary peritoneal macrophages of naive WT mice were pretreated with inhibitors for 1 h, followed by rhMANF treatment for 5 h. *Il10* mRNA expression was measured by RT-qPCR. **C** Phosphorylated and total p38 levels in primary liver macrophages from WT and *Manf*^Mye-/-^mice treated with APAP for 2 h. **D** Primary peritoneal macrophages derived from naive WT mice and *Manf*^Mye-/-^mice were treated with 100 ng/mL LPS or 2 μg/mL of rhMANF for 0.5 h. Phosphorylated and total p38 levels were analyzed by Western blotting. Data are presented as mean ± SD. *P < 0.05, **P < 0.01. *n* = 3–6 mice per group, 3 independent experiments.

To understand the mechanism by which MANF regulates IL-10 expression in macrophages, we screened several potential signaling pathways that are involved in IL-10 production in myeloid cells [27]. Our data revealed that rhMANF activated AKT and the MAPK family, including JNK, p38, and ERK1/2 in peritoneal macrophages of WT mice (Fig. 6A). We then treated peritoneal macrophages with rhMANF in combination with an AKT inhibitor and various MAPK inhibitors. Interestingly, only p38 inhibitor reduced IL-10 expression (Fig. 6B). Furthermore, the phosphorylation of p38 in liver macrophages of *Manf*^Mye-/-^ mice was decreased compared with WT mice after APAP treatment (Fig. 6C). In vitro experiments showed that when treated with LPS or rhMANF the phosphorylation of p38 was decreased in peritoneal macrophages of *Manf*^Mye-/-^ mice as compared with WT mice (Fig. 6D). These results suggested that MANF induced IL-10 expression by activating the p38 signaling pathway. To our surprise, rhMANF also induced *Tnfa*, *Il1b*, and *Il6* mRNA expression in naive mouse peritoneal macrophages and human macrophages, although this effect did not selectively depend on p38 MAPK (Supplementary Fig. S8B and Supplementary Fig. S9A). Considering that liver macrophages may be stimulated by DAMP-derived from APAP damaged hepatocytes in vivo, we further examined whether rhMANF could downregulate macrophage-mediated inflammatory response. We found that rhMANF pretreatment inhibited LPS-induced *Tnfa*, *Il1b*, and *Il6* expression, but not *Il10* (Supplementary Fig. S8C and Supplementary Fig. S9B).

### MANF promotes macrophage phagocytosis via the p38 signaling pathway

**Fig. 7:**
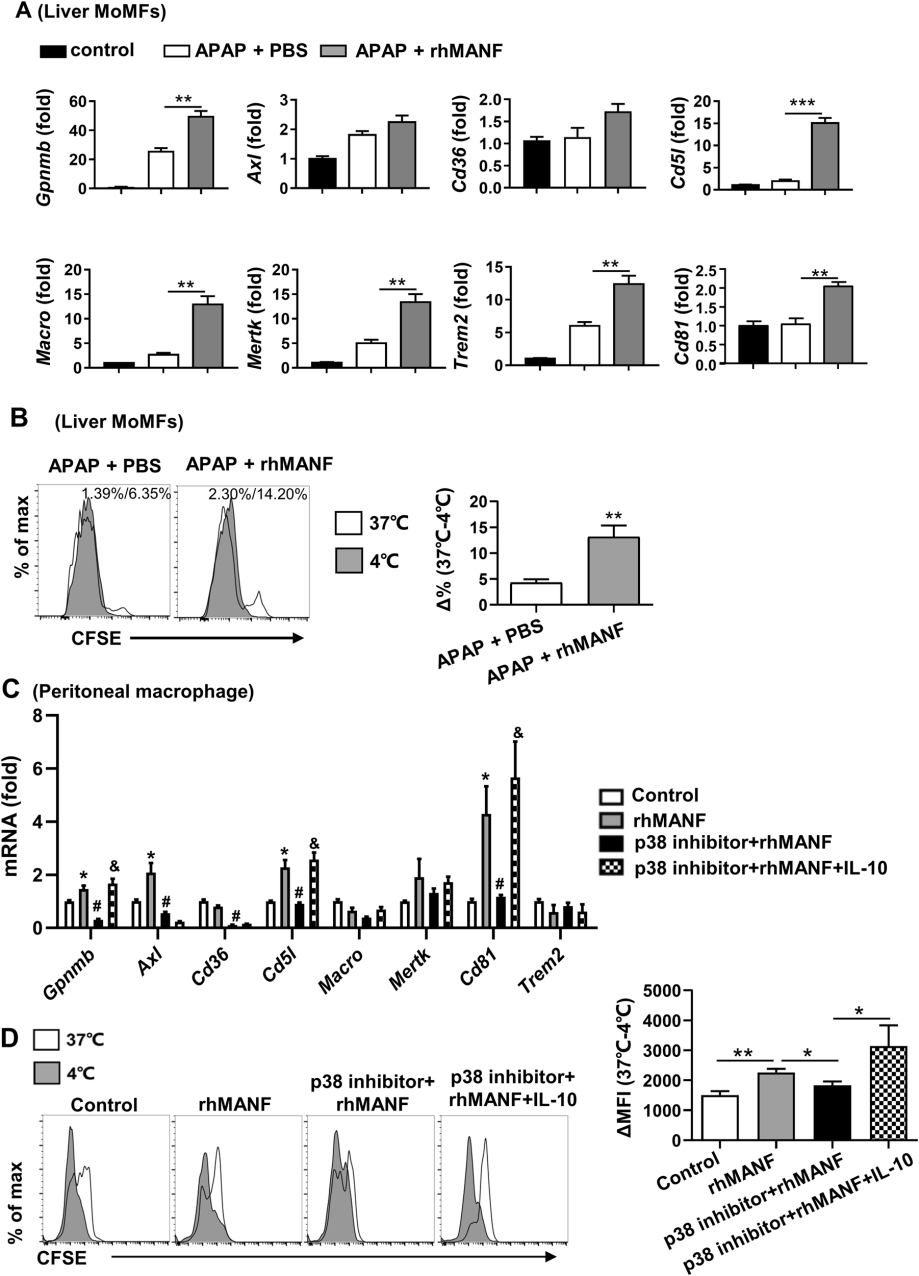
MANF enhances macrophage phagocytosis via the p38 MAPK activation. **A**, **B** Liver macrophages were isolated from naive (control), PBS/APAP- or rhMANF /APAP-treated WT mice 48 h after APAP injection. The phagocytosis-related gene levels in liver macrophages were measured. **B** Hepatocytes isolated from WT mice were treated with 20 μM dexamethasone for 24 h and then labeled with 1 μM CFSE for 15 min at 37 °C. Freshly isolated liver macrophages were incubated with CFSE-labeled hepatocytes at either 37 °C or 4 °C (control) for 30 min and analyzed by flow cytometry. Uptake of hepatocytes by MoMFs was shown as ∆ percentage (%) of CFSE positive MoMFs at 37–4 °C. **C**, **D** Peritoneal macrophages isolated from naive WT mice were pretreated with p38 inhibitor for 1 h, followed by rhMANF (2 μg/mL) with or without IL-10 (20 ng/mL) treatment for 5 h. **C** Phagocytosis-related gene expression was measured by RT-qPCR. *P < 0.05 compared with control; #<0.05 compared with rhMANF-treated group, & <0.05 compared with p38 inhibitor + rhMANF-treated group. **D** Uptake of hepatocytes by peritoneal macrophages were examined by flow cytometry and shown as ∆ mean fluorescence intensity (MFI) at 37–4 °C. Data are presented as mean ± SD. *n* = 4–6 mice per group, 3 independent experiments. *P < 0.05, **P < 0.01, ***P < 0.001.

It has been reported that phagocytosis drives the conversion of inflammatory macrophages to restorative macrophages [16, 18]. We analyzed the role of MANF on macrophage phagocytosis. A number of phagocytosis-related genes were examined in hepatic macrophages derived from APAP-treated WT mice and APAP/rhMANF-treated WT mice. As shown in Fig. 7A, rhMANF treatment upregulated the expression of *Gpnmb*, *Cd5l*, *Macro*, *Mertk*, *Trem2*, and *CD81*. The apoptotic hepatocyte uptake assay further demonstrated that rhMANF treatment greatly increased MoMF phagocytosis in vivo (Fig. 7B). Given that p38 has been reported to play a pivotal role in the ability of macrophages to phagocytose bacteria or particles [28, 29], we then examined whether the p38 signaling pathway is involved in the MANF-induced increase in macrophage phagocytosis. Our results showed that the p38 MAPK inhibitor decreased rhMANF-induced phagocytosis-related gene expression and phagocytosis of apoptotic hepatocytes in mouse peritoneal macrophages (Fig. 7C, D). It is worth noting that recombinant IL-10 restored the decreased phagocytic ability of macrophages caused by p38 inhibitor (Fig. 7C, D).

### MANF promotes IL-10 expression and phagocytosis in human monocytes through the p38 signaling pathway

**Fig. 8:**
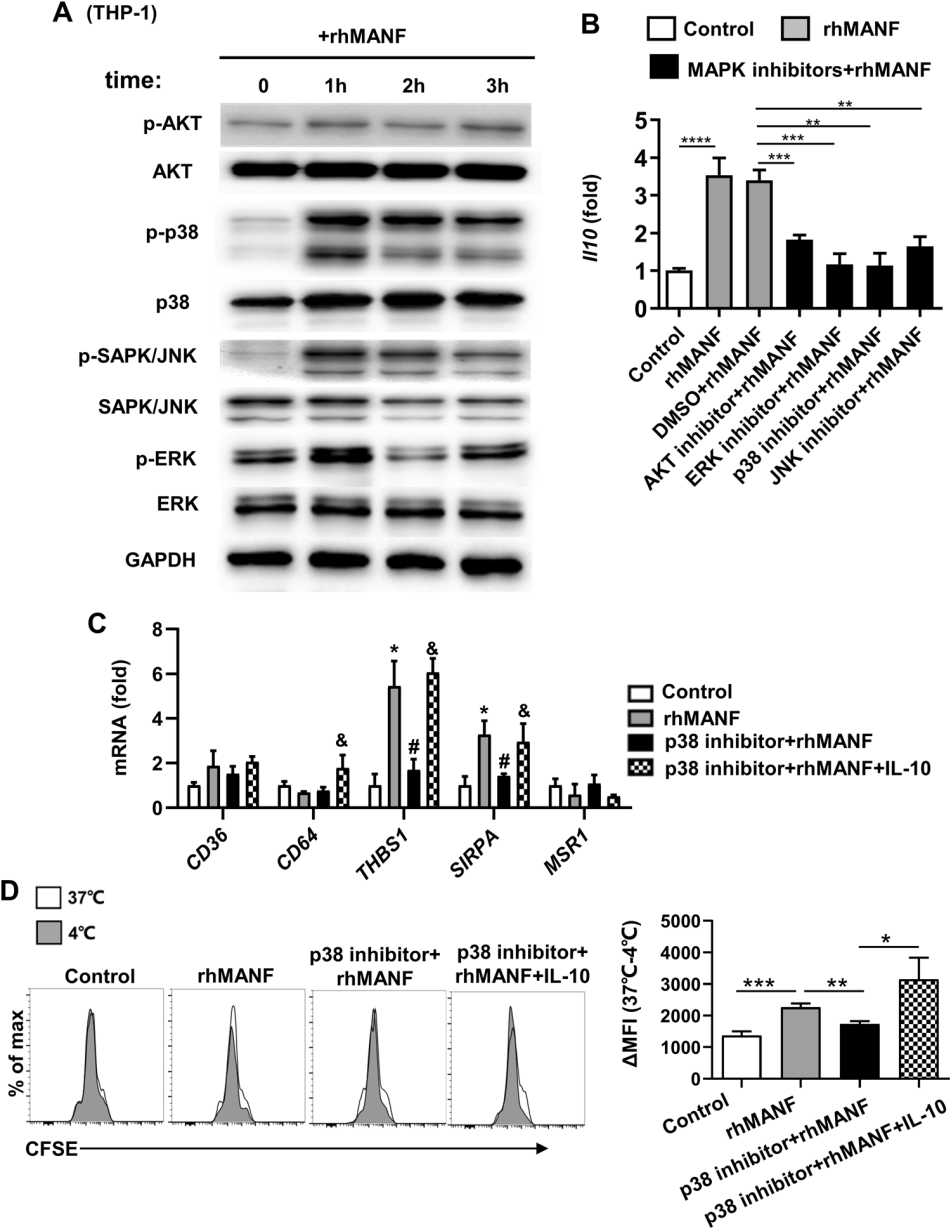
MANF induces IL-10 expression and phagocytosis in human monocytes via the p38 signaling pathway. **A** THP-1 cells were cultured with rhMANF (2 μg/mL) for the indicated times. Activation of various signaling pathways was measured by western blotting. **B** THP-1 cells were pretreated with various inhibitors for 1 h, followed by rhMANF (2 μg/mL) treatment for 5 h. *Il10* mRNA expression in THP-1 cells was measured by RT-qPCR. **C**, **D** THP-1 cells were cultured with p38 inhibitor for 1 h, followed by rhMANF (2 μg/mL) with or without IL-10 (20 ng/mL) treatment for 5 h. **C** Phagocytosis-related gene expression was measured by RT-qPCR. *P < 0.05 compared with control; #<0.05 compared with rhMANF-treated group, & <0.05 compared with p38 inhibitor + rhMANF-treated group. **D** Uptake of CFSE-labeled LO_2_ cells by THP-1 cells were examined by flow cytometry and shown as ∆ mean fluorescence intensity at 37–4 °C. Data are presented as mean ± SD. *n* = 3/group, 3 independent experiments. *P < 0.05, **P < 0.01.

Consistent with the above findings, rhMANF treatment also activated AKT, JNK, p38, and ERK1/2 MAPK in human THP-1 monocytes (Fig. 8A). All of the kinase inhibitors including p38 inhibitor, significantly reduced IL-10 expression levels in THP-1 cells (Fig. 8B). In addition, p38 inhibitor decreased rhMANF-induced phagocytosis-related gene expression and the phagocytosis of apoptotic hepatocytes in THP-1 cells (Fig. 8C, D). Moreover, IL-10 restored the decreased phagocytic ability of THP-1 cells caused by p38 inhibitor (Fig. 8C, D).

## Discussion

Recently, MANF has been described to have a function in regulating immune homeostasis in aging and protecting against liver damage [22]. However, the role of MANF in acute liver injury is still largely unclear. In this study, we revealed a function of MANF in mediating tissue repair after AILI by limiting MoMF infiltration and triggered inflammatory Ly6C^hi^ monocytes transition to pro-resolution Ly6C^low^ MoMFs. Mechanistically, the interaction of MANF with p38 resulted in increased IL-10 expression and enhanced phagocytosis in macrophages. The fact that rhMANF treatment was sufficient to regulate MoMF infiltration, macrophage programming, and liver repair, suggesting the therapeutic potential of rhMANF in treating APAP overdose-induced liver injury and other liver diseases.

We demonstrated that MANF was highly elevated in DILI patients and in an AILI mouse model. Both hepatic macrophages and stressed hepatocytes expressed MANF in response to liver injury, however, the increase of MANF expression in macrophages was higher than that in hepatocytes. Furthermore, *Manf*^Mye-/-^ mice showed increased inflammation and liver injury at the late phase after APAP injection, whereas no increase in inflammation and liver injury was observed in *Manf*^Hep-/-^ mice compared to WT mice, suggesting that the autocrine immunomodulatory function of MANF in myeloid cells was essential for the resolution of APAP-induced liver injury. Notably, rhMANF treatment was sufficient to promote liver repair in both WT mice and *Manf*^Mye-/-^ mice. A recent study showed that MANF deficiency in Cx3cr1-expressing monocytes or macrophages resulted in liver damage and inflammation, however, hepatocyte-specific ablation of MANF did not result in increased immune activation and liver damage [22], which supported our findings.

Our data showed that MANF could extensively inhibit monocyte infiltration during the resolution phase. *Manf*^Mye-/-^ mice had increased levels of CCL2 and CCL3 in the liver. MANF-deficient CD115^+^ monocytes expressed higher surface levels of CCR2, which augmented cell mobilization and influx, possibly explaining the increased monocyte infiltration during the late phase compared with WT mice. During AILI, bone marrow-derived monocytes are recruited in the damaged liver tissue [30]. Generally, the main purpose of these monocytes is to remove necrotic cell debris and promote liver repair, but dysregulated inflammation serves as a detrimental factor in many liver diseases by exacerbating liver damage [31]. Blocking recruitment of infiltrating Ly-6C^hi^ monocytes during the fibrosis regression phase accelerates resolution of toxic and metabolic liver fibrosis [32]. In this study, rhMANF treatment inhibited the extinctive monocyte infiltration in the liver, which might partially contribute to the accelerated liver injury regression induced by rhMANF. Future studies will need to determine how MANF influences CCR2/CCL2 expression. It will also be interesting to discern whether neutrophils which are also deficient in MANF, contribute to the heightened influx of monocytes in *Manf*^Mye-/-^ mice.

On the other hand, MANF orchestrated liver repair by triggering hepatic macrophage transition into a reparative phenotype. First, MANF promoted MoMFs to produce IL-10, which is an anti-inflammatory cytokine and was expressed much higher in Ly6C^low^ macrophages than that in Ly6C^hi^ macrophages [33]. Deficiency of the IL-10 gene in mice resulted in higher liver injury after APAP injection by enhancing the levels of NO and proinflammatory cytokines [34]. In our study, *Manf*^Mye-/-^ mice showed decreased levels of *Il10* in the liver especially in hepatic macrophages. Treatment of *Manf*^Mye-/-^ mice with recombinant IL-10 protected against AILI. Second, MANF promoted macrophage phagocytosis. rhMANF treatment upregulated the expression of phagocytosis-related genes in macrophages thus enhancing macrophage phagocytosis. It has been reported that induction of phagocytic behavior enhances the restorative phenotype of macrophages [16, 18]. Phagocytosis of necrotic hepatocytes by macrophages is important for liver repair and regeneration [35].

Mechanically, our results demonstrated that MANF promoted macrophage transition into a reparative phenotype, possibly by interacting with p38 MAPK. The p38 MAPK pathway is well known for its role in transducing stress signals from environments such as pathogens, heat shock, growth factors, and cytokines [36]. In this study, MANF enhanced p38 phosphorylation in macrophages. Previous studies have demonstrated that p38 activation contributed to IL-10 production in TLR-stimulated macrophages [37, 38], and enhanced the ability of macrophages to phagocytose bacteria [29, 39]. In addition, p38/MAPKAP kinase 2 (MK2) has been shown to be involved in the repolarization of M2 tumor-associated macrophages [40, 41]. A recent study showed that MANF protected mice against high-fat diet–induced obesity by promoting adipose browning via p38 MAPK pathway [42]. Here, our data showed that MANF promoted IL-10 expression and phagocytosis in macrophages via p38 MAPK. Notably, our in vitro experiments showed that IL-10 restored the decreased phagocytic ability of macrophages caused by p38 inhibitor, which suggested that MANF-induced IL-10 could further enhance the ability of macrophages to phagocytose necrotic hepatocytes.

More recently, a series of studies suggested that inflammatory Ly6C^hi^ monocytes transition into restorative Ly6C^low^ macrophages at the site of injury [12, 14, 16, 30, 43]. Identifying the molecular mechanisms underlying the phenotypic switch might serve as a starting point for new translational approaches. In this study, we provided evidence that myeloid-derived MANF was an important endogenous protective factor that promoting resolution of AILI. The rhMANF has therapeutic potential at the late phase of AILI. The protective role of MANF was mediated by inhibiting the extensive accumulation of monocytes in the liver and promoting macrophage transition into a reparative phenotype. The MANF and p38 were critical for shaping the phenotype and function of MoMFs.

## Supplementary information

Reproducibility Checklist

Mesencephalic astrocyte-derived neurotrophic factor reprograms macrophages to promote regression of acetaminophen-induced acute liver injury via p38 MAPK pathway

## Data Availability

The data that support the findings of this study are available from the corresponding author upon reasonable request.
