# Supplementary material for: Mesencephalic astrocyte-derived neurotrophic factor reprograms macrophages to ameliorate acetaminophen-induced acute liver injury via p38 MAPK pathway
Source: Cell Death Dis. 2022 Feb 2;13(2):100. doi: 10.1038/s41419-022-04555-9 (PMC8810950; doi:10.1038/s41419-022-04555-9)
Supplement: Supplementary file 2 — Mesencephalic astrocyte-derived neurotrophic factor reprograms macrophages to promote regression of acetaminophen-induced acute liver injury via p38 MAPK pathway [file 41419_2022_4555_MOESM2_ESM.doc]

***Supporting Materials***

**Mesencephalic astrocyte-derived neurotrophic factor reprograms macrophages to promote regression of acetaminophen-induced acute liver injury via p38 MAPK pathway**

Xin Hou^1, 2*^, Qi Liu^1, 3*^, Yimin Gao^1, 3*^, Liang Yong^3^, Huiyuan Xie^3^, Wenting Li^4^, Yuping Zhou^1^, Jun Liu^3^, Lijie Feng^3^, Long Xu^3^, Yuxian Shen^3^ & Hua Wang^5^

^1^The Affiliated Hospital of Medicine School, Ningbo University, Ningbo, China

^2^School of Medicine, Ningbo University, Ningbo, China

^3^School of Basic Medical Sciences, Anhui Medical University, Hefei, China

^4^The First Affiliated Hospital, Division of Life Sciences and Medicine, University of Science and Technology of China, Hefei, China

^5^Department of Oncology, the First Affiliated Hospital of Anhui Medical University, Hefei, China.

^*^ These authors contributed equally to this work.

Supporting Table S1: Information of NALDF patients for cohort study

| **Variables** | **DILI patients (n=24)** |
| --- | --- |
| ALT (IU/L) | 313.7 ± 358.8 |
| AST (IU/L) | 216.8 ± 237.9 |
| ALP (IU/L) | 218.5 ± 204.7 |
| Monocytes (%) | 9.8 ± 3.8 |
| Monocytes (×10^9^/L) | 0.5 ± 0.3 |
| Total bilirubin (μmol/L) | 123.0 ± 141.1 |

**Supporting Table S2: RT-qPCR primer** sequences

| **Gene** | **Human/Mouse** | **Primer** | **Sequence(5,-3,)** |
| --- | --- | --- | --- |
| MANF | Mouse | Forward | CACCAGCCACTATTGAAGAAGA |
|  |  | Reverse | AGCATCATCTGTGGCTCCAA |
| TNF-α | Mouse | Forward | ACTGGCAGAAGAGGCACTC |
|  |  | Reverse | CTGGCACCACTAGTTGGTTG |
| IL-1β | Mouse | Forward | CTGAACTCAACTGTGAAATGC |
|  |  | Reverse | TGATGTGCTGCTGCGAGA |
| IL-6 | Mouse | Forward | ACACATGTTCTCTGGGAAATCGT |
|  |  | Reverse | AAGTGCATCATCGTTGTTCATACA |
| IL-10 | Mouse | Forward | GCTCTTACTGACTGGCATGAG |
|  |  | Reverse | CGCAGCTCTAGGAGCATGTG |
| Ym-1 | Mouse | Forward | TCTGAAAGACAAGAACACTGAGC |
|  |  | Reverse | GCAGGTCCAAACTTCCATCC |
| Arg-1 | Mouse | Forward | AACACGGCAGTGGCTTTAACC |
|  |  | Reverse | GGTTTTCATGTGGCGCATTC |
| CCL2 | Mouse | Forward | CCAGCAAGATGATCCCAATG |
|  |  | Reverse | TACGGGTCAACTTCACATTC |
| CCL3 | Mouse | Forward | GATTCCACGCCAATTCATCG |
|  |  | Reverse | AGGCATTCAGTTCCAGGTCA |
| Gpnmb | Mouse | Forward | CATTCCCATCTCGAAGGTGAAA |
|  |  | Reverse | AAATGGCAGAGTCGTTGAGGA |
| Axl | Mouse | Forward | ATGGCCGACATTGCCAGTG |
|  |  | Reverse | CGGTAGTAATCCCCGTTGTAGA |
| CD36 | Mouse | Forward | ATGGGCTGTGATCGGAACTG |
|  |  | Reverse | GTCTTCCCAATAAGCATGTCTCC |
| CD51 | Mouse | Forward | GATCGTGTTTTTCAGAGTCTCCA |
|  |  | Reverse | TGCAGTCAACCCCTTGAATAAG |
| Macro | Mouse | Forward | ACAGAGCCGATTTTGACCAAG |
|  |  | Reverse | CAGCAGTGCAGTACCTGCC |
| Mertk | Mouse | Forward | CAGGGCCTTTACCAGGGAGA |
|  |  | Reverse | TGTGTGCTGGATGTGATCTTC |
| Trem2 | Mouse | Forward | CTGGAACCGTCACCATCACTC |
|  |  | Reverse | CGAAACTCGATGACTCCTCGG |
| CD81 | Mouse | Forward | GTGGAGGGCTGCACCAAAT |
|  |  | Reverse | GACGCAACCACAGAGCTACA |
| Actin | Mouse | Forward | GGCTGTATTCCCCTCCATCG |
|  |  | Reverse | CCAGTTGGTAACAATGCCATGT |
| MANF | Human | Forward | CAGCCACCAAAATCATCAATGAGG |
|  |  | Reverse | TCCACTGTGCTCAGGTCGATCT |
| IL-10 | Human | Forward | TCTCCGAGATGCCTTCAGCAGA |
|  |  | Reverse | TCAGACAAGGCTTGGCAACCCA |
| TNF-α | Human | Forward | CTCTTCTGCCTGCTGCACTTTG |
|  |  | Reverse | ATGGGCTACAGGCTTGTCACTC |
| IL-1β | Human | Forward | CCACAGACCTTCCAGGAGAATG |
|  |  | Reverse | GTGCAGTTCAGTGATCGTACAGG |
| IL-6 | Human | Forward | AAATTCGGTACATCCTCGACGGCA |
|  |  | Reverse | AGTGCCTCTTTGCTGCTTTCACAC |
| IL-12α | Human | Forward | TGCCTTCACCACTCCCAAAACC |
|  |  | Reverse | CAATCTCTTCAGAAGTGCAAGGG |
| IL-12β | Human | Forward | GACATTCTGCGTTCAGGTCCAG |
|  |  | Reverse | CATTTTTGCGGCAGATGACCGTG |
| CCL2 | Human | Forward | AGAATCACCAGCAGCAAGTGTCC |
|  |  | Reverse | TCCTGAACCCACTTCTGCTTGG |
| CXCL1 | Human | Forward | AGCTTGCCTCAATCCTGCATCC |
|  |  | Reverse | TCCTTCAGGAACAGCCACCAGT |
| CXCL2 | Human | Forward | GGCAGAAAGCTTGTCTCAACCC |
|  |  | Reverse | CTCCTTCAGGAACAGCCACCAA |
| CXCL8 | Human | Forward | GAGAGTGATTGAGAGTGGACCAC |
|  |  | Reverse | CACAACCCTCTGCACCCAGTTT |
| CD36 | Human | Forward | CAGGTCAACCTATTGGTCAAGCC |
|  |  | Reverse | GCCTTCTCATCACCAATGGTCC |
| CD64 | Human | Forward | GCATGGGAAAGCATCGCTAC |
|  |  | Reverse | GCAAGAGCAACTTTGTTTCACA |
| Thbs1 | Human | Forward | GCTGGAAATGTGGTGCTTGTCC |
|  |  | Reverse | CTCCATTGTGGTTGAAGCAGGC |
| Sirpα | Human | Forward | GGCCTCAACCGTTACAGAGAA |
|  |  | Reverse | GTTCCGTTCATTAGATCCAGTGT |
| Msr1 | Human | Forward | TGCACAAGGCAGCTCACTTTGG |
|  |  | Reverse | GTGCAAGTGACTCCAGCATCTTC |
| GAPDH | Human | Forward | GTCTCCTCTGACTTCAACAGCG |
|  |  | Reverse | ACCACCCTGTTGCTGTAGCCAA |


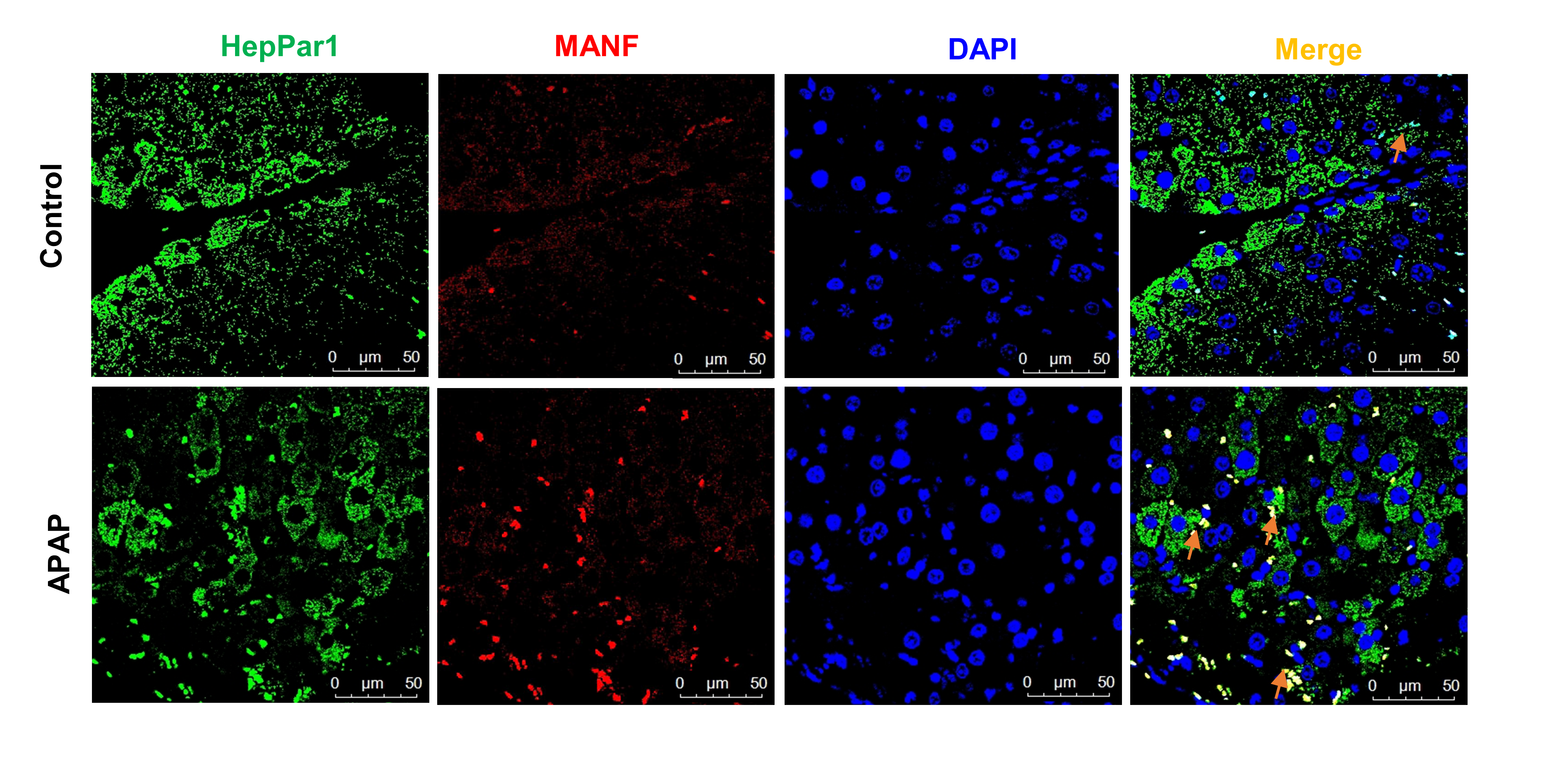


Supporting Fig. S1. **MANF expression in hepatocytes were increased mildly after APAP injection**. Immunofluorescence staining for HepPar1 (green), MANF (red) and DAPI for nuclei (blue) in the liver of WT mice with or without APAP injection. Orange arrows point to MANF^+^ hepatocytes.


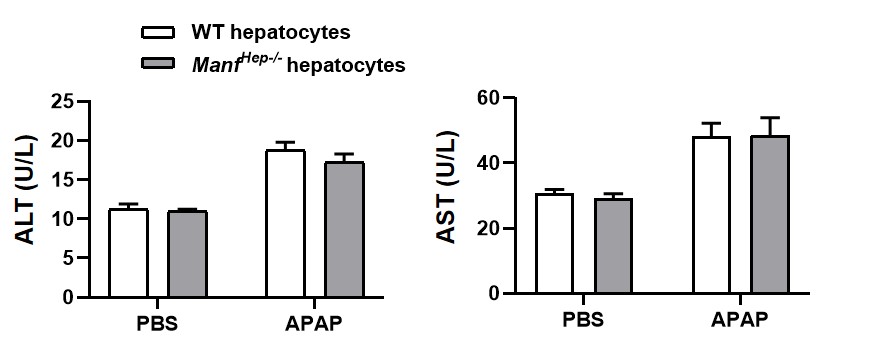


**Supporting Fig. S2. MANF does not directly affect APAP induced hepatocyte death in cultured hepatocytes.** Primary hepatocytes were isolated from WT and *Man*^Hep-/-^mice, and then treated with 5mM APAP. Supernatant was collected 24 hours after APAP treatment for ALT and AST measurement.


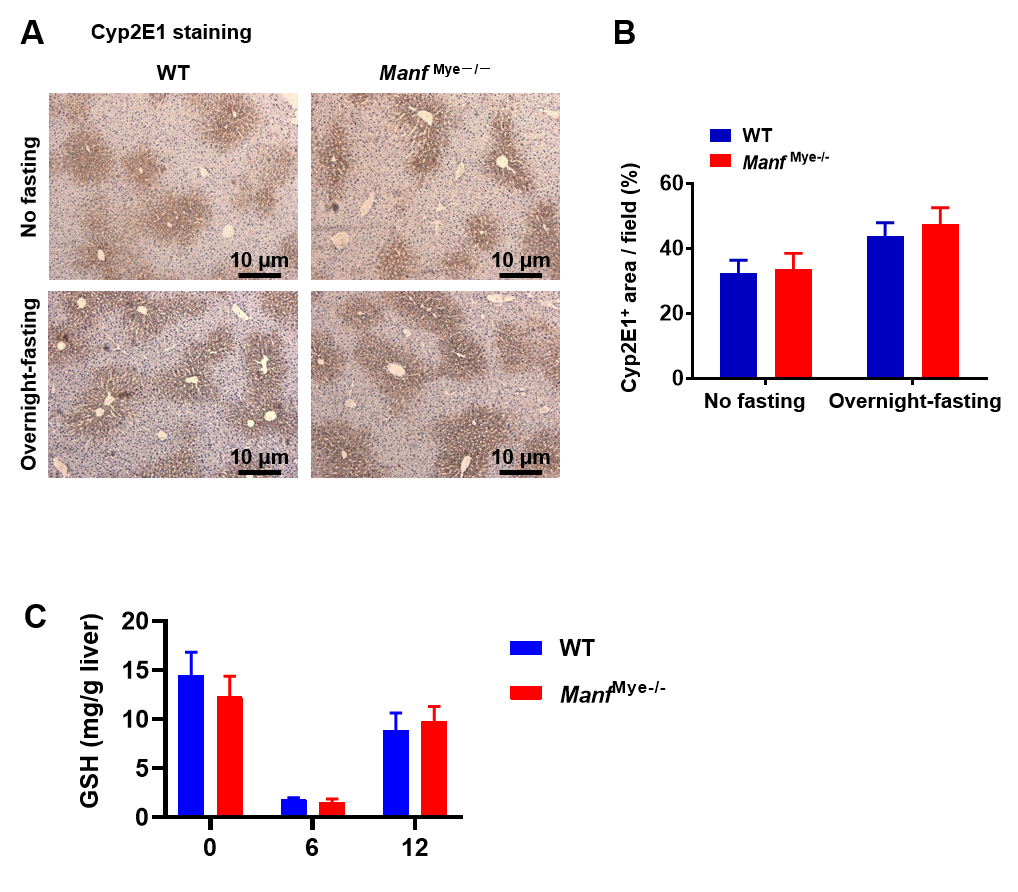


**Supporting Fig. S3. Myeloid specific deletion of Mesencephalic astrocyte-derived neurotrophic factor (MANF) does not influence bioactivation of APAP.** (A, B) WT and *Manf*^Mye-/-^mice were overnight fasted or no fasted. The levels of Cyp2e1 protein expression were detected by immunohistochemical staining. Quantification using image J. n = 6 mice per group. (C) Fasted WT and *Manf*^Mye-/-^mice were injected with 300 mg/kg of APAP. Glutathione (GSH) levels in liver homogenates were measured at 0 (before treatment), 6, and 12 hours after APAP challenge by an Assay Kit (Solarbio, Beijing, China). Values represent means ± SD.


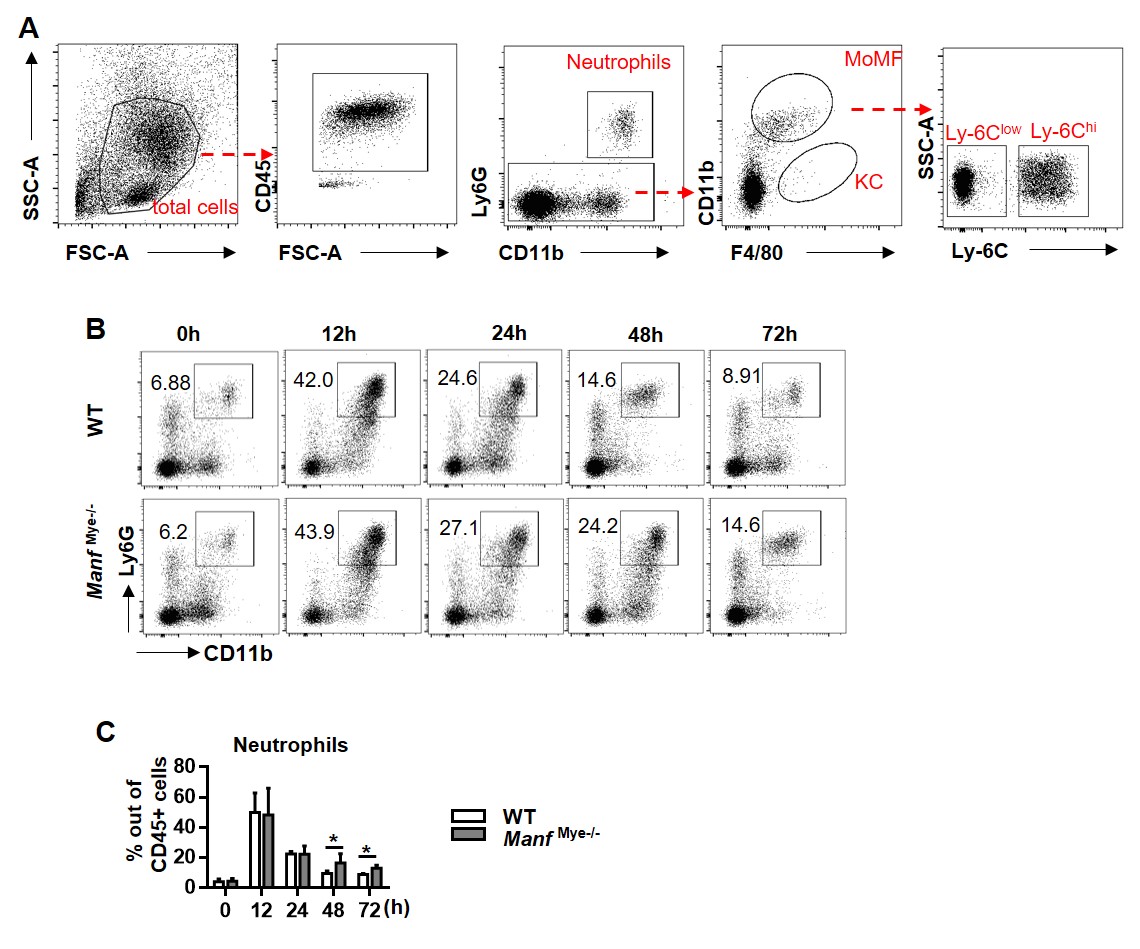


**Supporting Fig. S4.** (A) Gating strategy for detection of liver neutrophils, infiltrating monocyte-derived macrophages (MoMFs), Ly6C^hi^ and Ly6C^low^ MoMFs. (B, C) Fasted WT and *Manf*^Mye-/-^mice were injected with 300 mg/kg of APAP, and measured at the indicated time points. Representative flow cytometric plots (B) and the statistical quantification of hepatic neutrophils (Ly6G^+^CD11b^+^) (C). Data are presented as mean ± SD (n≥9). **P* < 0.05 (unpaired Student’s t-test).


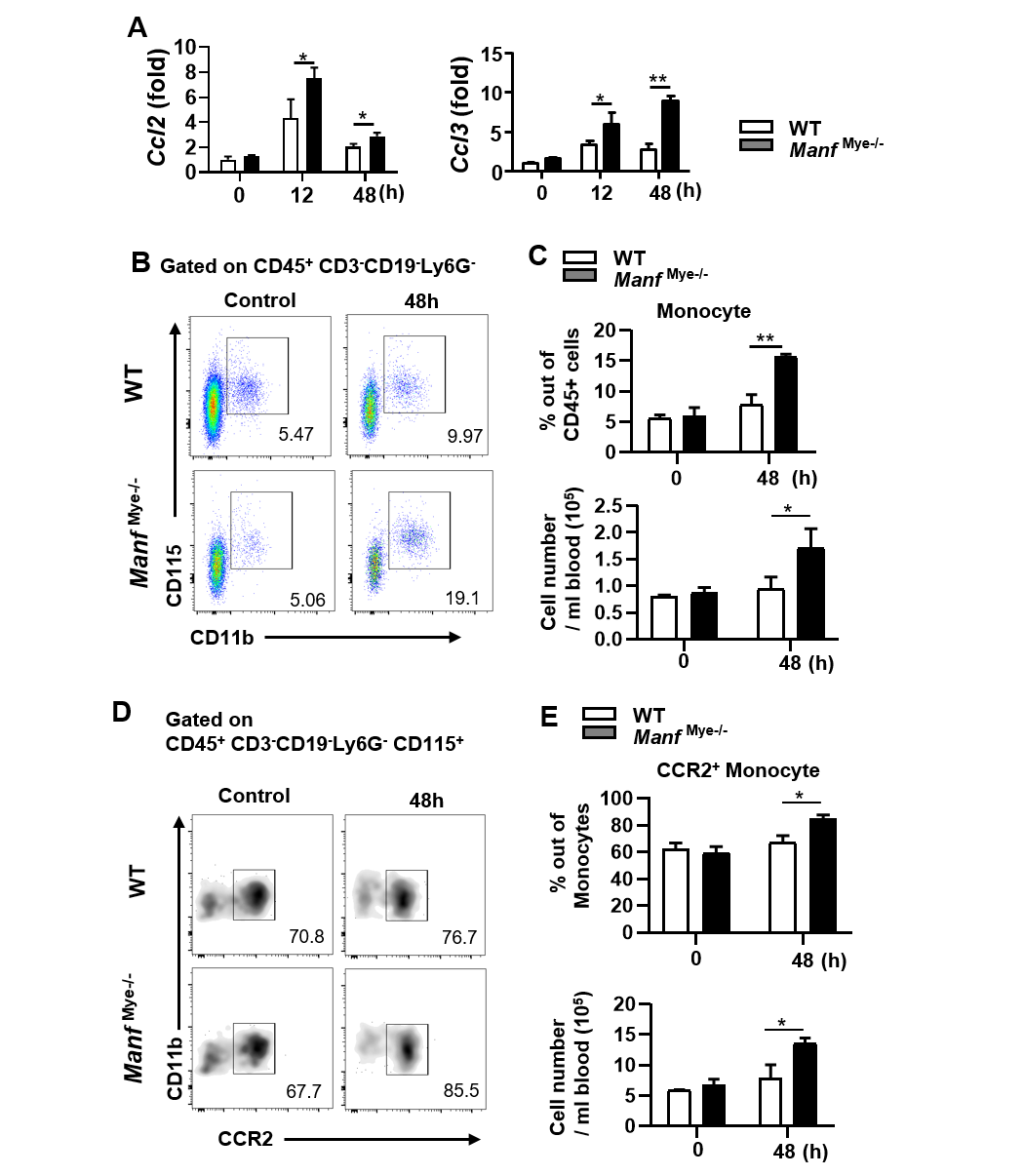


**Supporting Fig. S5.** **MANF deficiency promotes C–C chemokine receptor (CCR2)/CCL2-mediated monocyte recruitment into the liver.** WT and *Manf*^Mye-/-^ mice were intraperitoneally injected with APAP (300 mg/kg). (A) The mRNA expression of *Ccl2* and *Ccl3* in the liver was measured by qPCR. (B-E) Peripheral blood was obtained 48 h after APAP treatment. Representative flow cytometric plots and the statistical quantification of CD115^+^ monocytes in the peripheral blood of WT and *Manf*^Mye-/-^ mice. (D) Representative dot plot of flow cytometric staining for CCR2 expression in circulating CD115^+^monocytes. (E) Quantification of CCR2^+^ monocytes in peripheral blood of WT and *Manf*^Mye-/-^ mice by percentage and absolute numbers. Results are presented as mean ± SD, **P* < 0.05, ***P* < 0.01. n=5 per group.


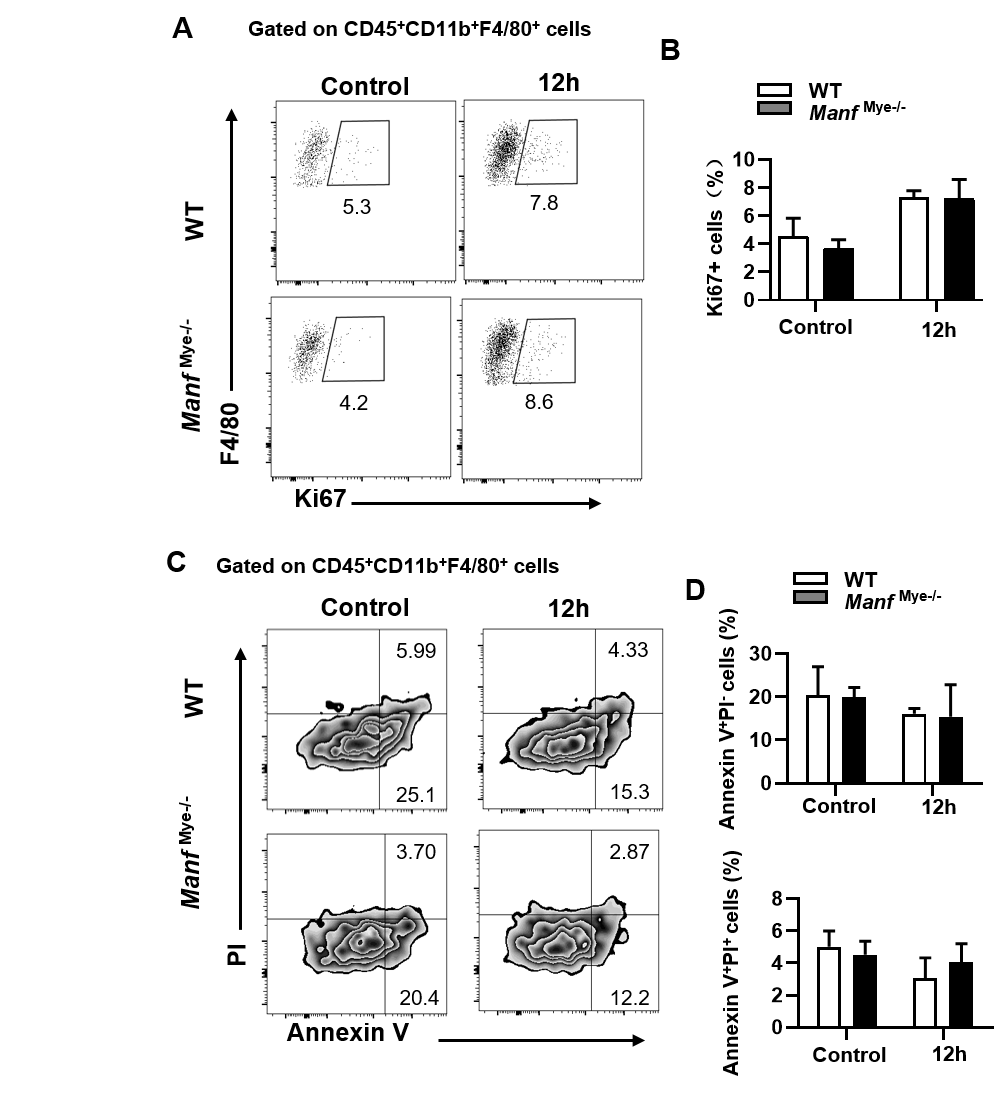


**Supporting Fig. S6.** WT and *Manf*^Mye-/-^ mice were intraperitoneally injected with APAP (300 mg/kg), and liver tissues were obtained 12 hours later. (A) Flow cytometry analysis of Ki67^+^CD45^+^CD11b^+^F4/80^+^ cells in liver tissues of WT and *Manf*^Mye-/-^ mice. (B) Percentages of Ki67^+^ cells out among CD45^+^CD11b^+^F4/80^+^ cells in liver tissues of WT and *Manf*^Mye-/-^ mice. (C) Flow cytometry analysis of CD45^+^CD11b^+^F4/80^+^ cell death using dual Annexin V-FITC and propidium iodide (PI) staining. (D) Graphical summaries showing the percentages of Annexin V^+^PI^+^ cells and Annexin V^+^PI^-^ cells of CD45^+^CD11b^+^F4/80^+^ cells in liver tissues of WT and *Manf*^Mye-/-^ mice.


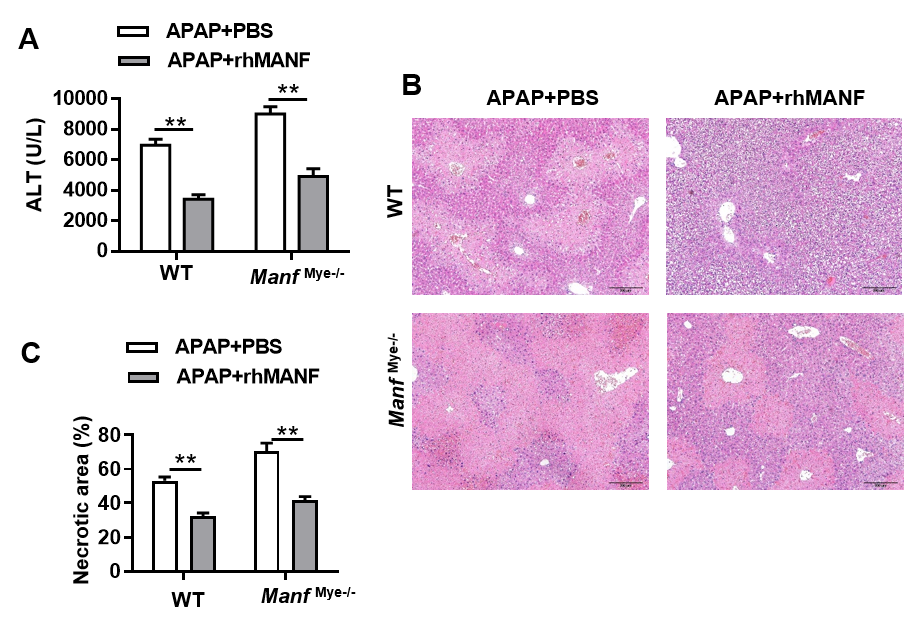


**Supporting Fig. S7. rhMANF accelerates AILI resolution.** WT mice and *Manf*^Mye-/-^mice were intravenously injected with rhMANF (1.5 mg/kg) at the same time as APAP administration. (A) Serum ALT activities were determined at 24 hours after APAP administration. (B) Representative H&E-stained liver sections at 24 hours after APAP treatment (magnification × 40). (D) Necrotic areas were quantified by ImageJ software. Data are presented as mean ± SD, n = 8-10 mice per group from two independent experiments. ***P* < 0.01.


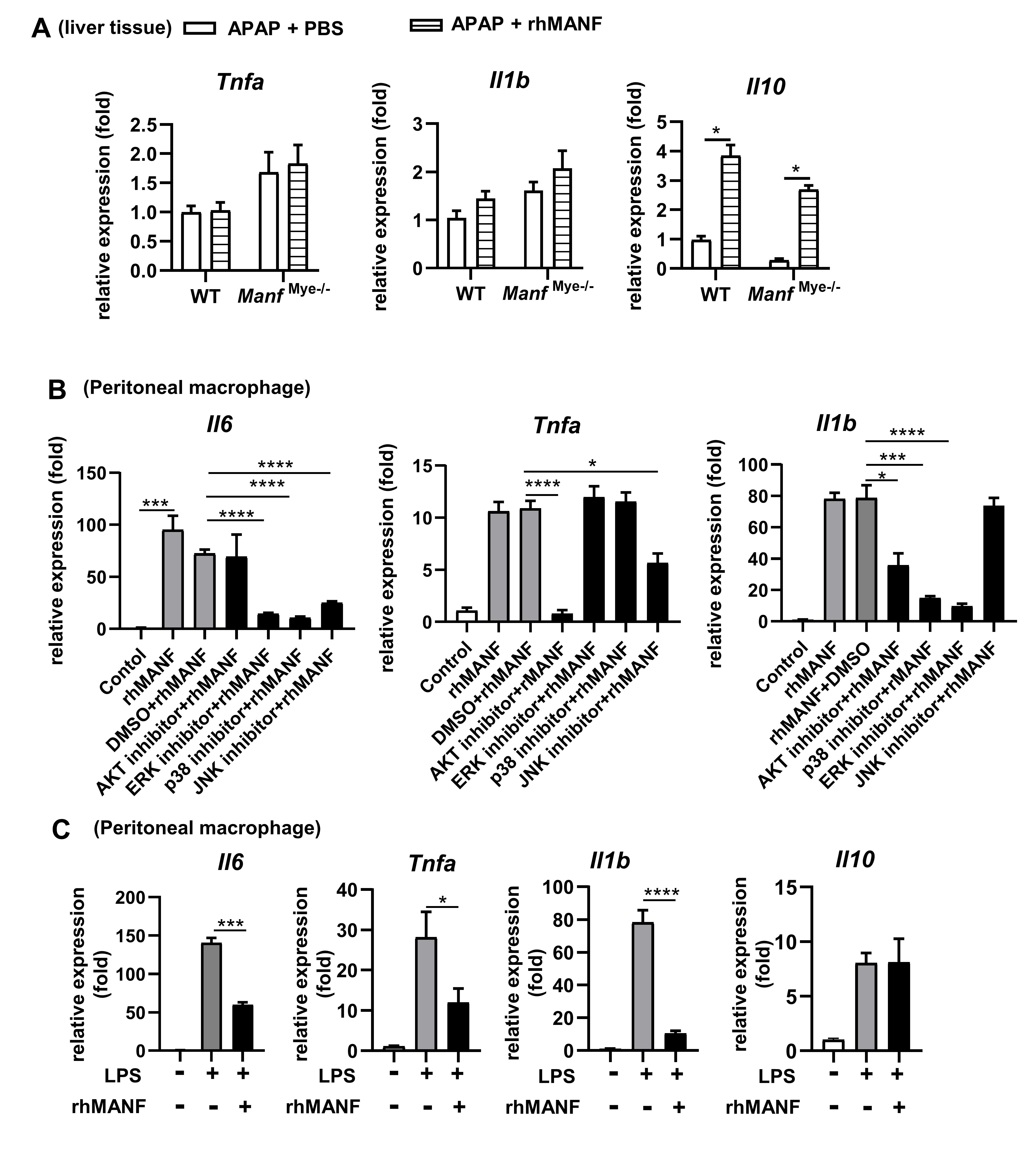


**Supporting Fig. S8.** **The role of rhMANF in mouse macrophage cytokine expression.** (A) WT mice and *Manf*^Mye-/-^mice were intravenously injected with rhMANF (1.5 mg/kg) at the same time as APAP administration. *Tnfa*, *Il1b*, and *Il10* mRNA expression in liver tissues were measured by RT-qPCR. (B) Primary peritoneal macrophages of naïve WT mice were pretreated with inhibitors for 1 hour, followed by rhMANF (2 μg/mL) treatment for 5 hours. *Il6, Tnfa*, and *Il1b* mRNA expression were measured by RT-qPCR. (C) Primary peritoneal macrophages of naïve WT mice were treated with rhMANF (2 μg/mL) for 10 hours, and then exposed to 100 ng/mL of LPS for 6 hours. Gene expression levels were measured by RT-qPCR. Data are presented as mean ± SD, **P* < 0.05, ****P* < 0.001, **** *P* < 0.0001.


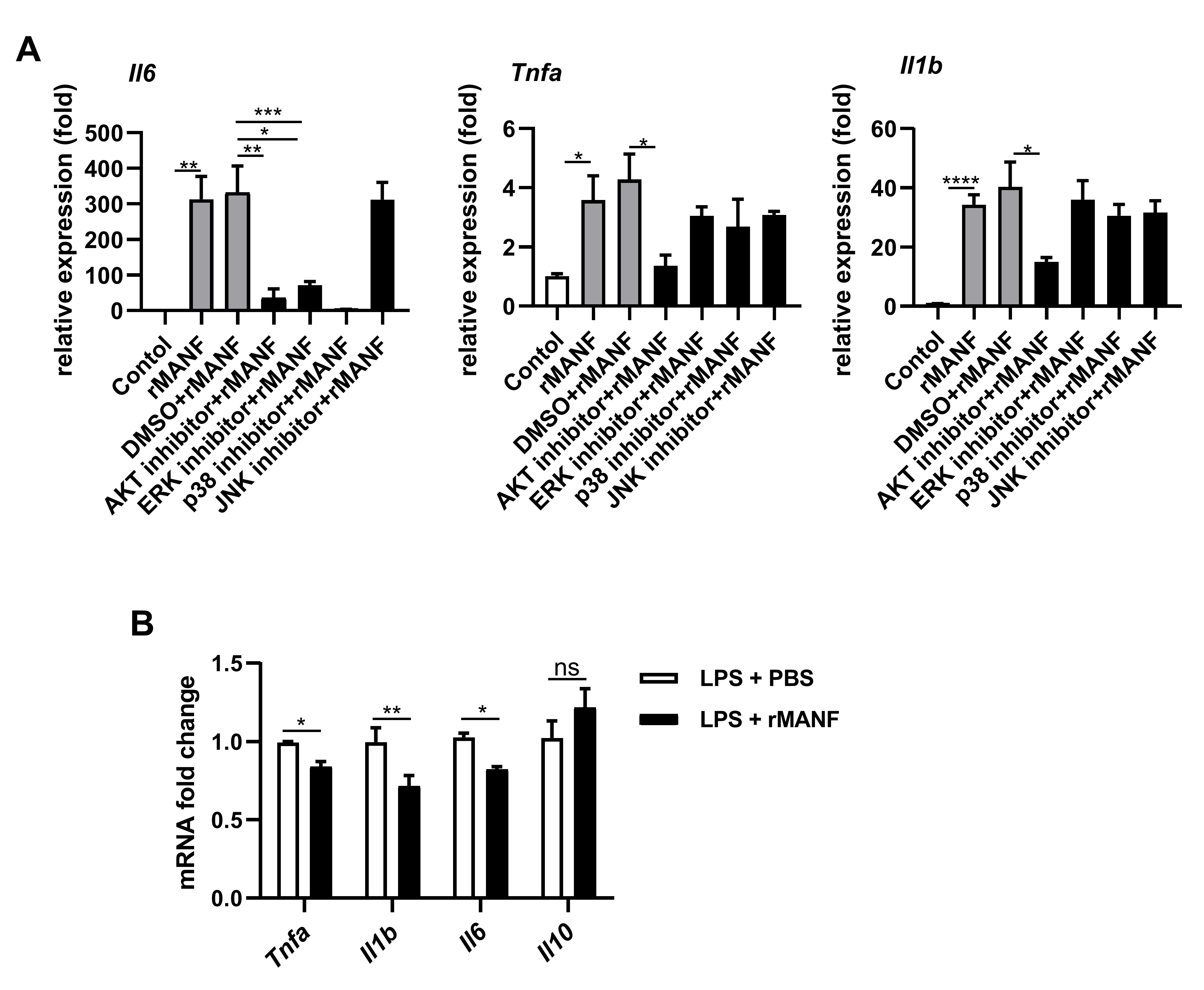


**Supporting Fig. S9.** **The role of rMANF in human macrophage cytokine expression.** (A) THP-1 cells were pretreated with various inhibitors for 1 hour, followed by rMANF (2 μg/mL) treatment for 5 hours. *Il6, Tnfa*, and *Il1b* mRNA expression in THP-1 cells was measured by RT-qPCR. (B) THP-1 cells were treated with rMANF (2 μg/mL) for 10 hours, and then exposed to 100 ng/mL of LPS for 6 hours. Gene expression levels were measured by RT-qPCR. Data are presented as mean ± SD, **P* < 0.05, ***P*<0.01, ****P* < 0.001, **** *P* < 0.0001.
